# Bioinspired Fabrication of Polyurethane/Regenerated Silk Fibroin Composite Fibres with Tubuliform Silk-Like Flat Stress–Strain Behaviour

**DOI:** 10.3390/polym10030333

**Published:** 2018-03-19

**Authors:** Harun Venkatesan, Jinlian Hu, Jianming Chen

**Affiliations:** Institute of Textiles & Clothing, The Hong Kong Polytechnic University, Hung Hom, Kowloon 999077, Hong Kong, China; venkatharun@gmail.com

**Keywords:** polyurethane, regenerated silk fibroin, composite fibre, tubuliform silk, flat tensile stress–strain curve

## Abstract

Tubuliform silk is one of the seven different types of spider silks, which is well known for its unique tensile behaviour with Flat Tensile Stress–Strain (FTSS) curve. It is found that anisotropic microstructure of β-sheets is responsible for this property. In recent years, bioinspired approaches to engineer fibres supported by modern manufacturing systems have been attracting considerable interest. The present paper aims to investigate a strategy to biomimic the FTSS behaviour of tubuliform silk in synthetic polymer composite fibres by blending polyurethane (PU) and regenerated silk fibroin (RSF) at different ratios. Wet spinning of composite fibres results in the reconstruction of β-sheets in the synthetic fibre matrix. PU/RSF composite fibre at a ratio of 75/25 produce a tensile curve with FTSS characteristics. Secondary structural changes in RSF and interchain directions of β-sheets within the fibre are studied using Fourier Transform Infra-red (FTIR) spectroscopy and Transmission Electron Microscopy (TEM), respectively. Interestingly, results of TEM patterns confirm transverse anisotropic properties of RSF β-sheets. The composite fibres also display tuneable mechanical properties with respect to RSF contents.

## 1. Introduction

Natural fibres often get their leverage from sophisticated microstructure organisation to achieve special functionalities. Chemical building blocks in biological materials are hierarchically organized in a way to fulfil the functional needs of organisms. The influence of microstructure in deciding the functional properties of natural fibres have been widely studied [1,2]. Molecular organisation of natural materials and their mechano-functionalities often exceed what is achievable using man-made approaches. For instance, a spider with seven different gland-spinneret complexes can synthesize a unique blend of structural polymers and produce seven different types of silk fibres with distinct set of functional properties through mechanical reeling process [3]. Spider silks are renowned for their excellent display of mechanical properties [4]. The impressive values of strength and elongation showed by dragline silk has attracted considerable attention and have been extensively studied. Following dragline silk, tubuliform or egg case silk is the second most characterized spider silk for their properties. Tubuliform silk is solely produced by orb-weaving female spiders during reproductive season and utilized to protect spider offspring from crushing, predators and climate changes [5]. Vollrath et al. [6] studied the stress–strain curves of *Nephila* golden spider silk and demonstrated a stress–strain behaviour of egg case silks is distinct from other types of spider silks. Stress–strain curves of egg case silk exhibited a unique mechanical behaviour with prominent yield accompanied by low modulus extension [3,7,8]. For dragline silks, the stress–strain curves begin with a small elastic region followed by a plastic region and failure occurs at strain hardening region. In egg case silk, the small elastic region is followed by extremely flat plastic hardening region. In both cases, the stress increases linearly with strain [9]. Barghout et al. revealed these specific mechanical behaviours of tubuliform silks were imparted by a multiaxial anisotropic microstructure, which was not observed for dragline silks [10]. The presence of twisted non-periodic lattices (NPL) parallel to the chain direction of the fibres was in contrast to dragline silks [11]. Non-periodic lattices are 70–500 nm sized voluminous ordered regions with less aligned molecules in dragline silk [12]. Based on these studies, the anisotropic arrangement of crystal like reinforcing regions and twisted NPL lattices regions present in tubuliform silk were suspected to be the reason for failure at plastic hardening region. Despite the above-mentioned studies, the mechanism for the mechanical properties of tubuliform silk remains unclear.

Surprisingly, this special ability of egg case silk to produce FTSS curves without strain hardening region are not exhibited by any other types of spider silks or even other commercial fibres. Normal fibres show a dramatic increase of tensile stress under higher elongation. Unlike these fibres, tubuliform silks can elongate at near-constant stress level after initial elastic modulus. This unique property of tubuliform silk facilitates the development of a new generation of fibres for functional textile and biomedical applications. Studies on spider silk’s microstructure and mechanical properties are inspiring new approaches in designing of advanced functional fibrous materials. Although considerable research has been devoted in developing recombinant spider silk fibres, rather less attention has been paid to synthetic polymers with similar mechanical behaviours [13,14,15,16,17,18]. Currently, artificial production of spider silk fibres remains a great challenge for two main reasons: (1) unlike silkworm silks, spiders cannot be farmed due to their cannibalistic nature; and (2) low yield and high production cost of spider silk proteins produced using recombinant technology. This situation demands alternative methods to produce spider silk like fibres. Understanding of the structure–function relationship of spider silk is a key step for developing spider silk inspired fibres. Polyurethanes are biodegradable polymers widely used for tissue engineering applications. Smart properties of PU have gained great attention over a decade for its innovative products in biomedical applications [19,20,21]. Various approaches of modifying chemically and physical blending approaches have suggested these modifications had influenced mechanical properties and shape memory behaviour of the material [22,23,24].

In the literature, there are several examples on PU and silk fibroin composite films [22,25,26,27,28], fibre [29] and hydrogels [30] based on physical blending and chemical modification methods. However, rare study is reported on the preparation of biomimetic Polyurethane/Regenerated silk fibroin (PU/RSF) composite fibres possessing egg case-silk-like mechanical properties. Task-specific fibres, egg case silks, exhibit the great potential as bandages for the diabetic and burnt patients with the unique flat tensile stress–strain (FTSS) mechanical behaviour. Thus, in this paper, we aim to develop a cost-effective biomaterial using a facile method with similar FTSS behaviour. This unique mechanical property is highly associated with the proportion, structure and orientation of β-sheet nanocrystals within silks. Polyurethanes as amorphous elastomers, are blended with regenerated silk fibroins in different ratios to control the proportion of β-sheet nanocrystals. The reconstruction of β-sheet nanocrystals is conducted by dissolving RSF in 1,1,1,3,3,3-hexafluoro-isopropanol (HFIP) and then exposing to alcoholic solvents for tuning the structure. Until now, this methodology to blend different concentration of regenerated silk fibroin with polyurethane has been applied only in studying films [22,25]. However, our study provides considerable insight into studying their respective fibre properties and their characteristic flat tensile stress–strain behaviour.

The physical properties of the amorphous PU fibres were modified using RSF with β-sheets formed. PU/RSF composites were wet-spun using methanol as a coagulant, by which the secondary structure of RSF can be regulated. Specifically, α-helix of RSF would be converted to β-sheets after spinning in the methanol bath. Interestingly, the crystals induced in the amorphous polymer matrix show anisotropic characteristics. The composite fibre with desired concentration of PU/RSF displayed stress–strain curves with flat plastic hardening region curves similar to tubuliform silk. In this way, we describe a method to biomimic the properties of the tubuliform silk in amorphous polyurethane fibres. In addition, the composite fibres also displayed tuneable mechanical properties with respect to RSF concentration. The morphology, secondary structure, thermal stability, mechanical properties and crystallinity of the PU/RSF composite fibres were investigated by SEM, FTIR, TGA, Tensile testing and TEM, respectively, to shed light on the study of mechanism of unique FTSS using a deformation model.

## 2. Materials and Methods

### 2.1. Synthesis of Polyurethane

**Figure 1:**
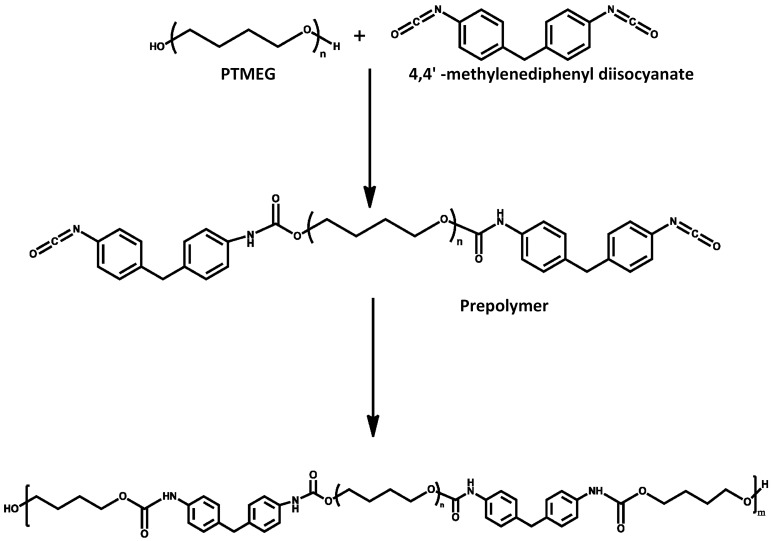
Schematic for synthesis of polyurethane.

PU was synthesized by bulk polymerization (see, Figure 1) according to previously described method using polytetramethylene ether glycol (PTMEG-650), 1,4-butanediol (BDO) and methylene diphenyl di-isocyanate (MDI) (raw materials purchased from Sigma Aldrich, St. Louis, MO, USA) [31]. The molar ratio of MDI, 1,4-butanediol and PTMEG was set to 1.5:0.5:1 and the content of soft chain segment and hard segment were 61% and 39%, respectively. Briefly, synthesis was carried out in a 500 mL round bottomed four necked flask equipped with a mechanical stirrer, thermometer and condenser. First, dehydration process of PTMEG for 2 h under reduced pressure at 90 °C was performed. Second, MDI and PTMEG were mixed and then stirred continuously for 1.5 h. Third, BDO was added and the reaction allowed to continue for 30 min. After the reaction, polymer was poured out into a Teflon plate and cured at 80 °C for 12 h inside a vacuum oven. Finally, the polymeric mould was crushed and pelletized using a single screw extruder.

### 2.2. Extraction of Silk Fibroin from B. mori Cocoons

**Figure 2:**
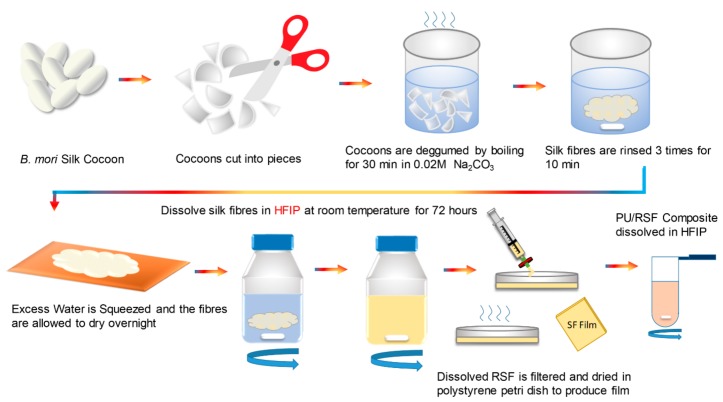
Schematic for PU/RSF polymer composite preparation procedure. Stages involved from cocoons to PU/RSF composite spinning dope.

Several studies reported the procedure for extraction of regenerated silk fibroin [32,33,34,35,36]. As shown in Figure 2, *B. mori* silk cocoons (purchased from Regent Science Industry Limited, Shenzhen, China) were cut into pieces and boiled in 0.02 M Na_2_CO_3_ (Sigma Aldrich, Saint Louis, USA) aqueous solution for 30 min. After boiling, silk was rinsed using ultrapure water for 10 min and repeated 3 times to remove residual sericin. After rinsing, excess water was squeezed out from the degummed silk and the fibre was spread in aluminium foil and allowed to dry overnight under a fume hood. Later, the dried fibres were dissolved in HFIP at room temperature for 72 h under continuous stirring. After dissolving, RSF was filtered using a PTFE syringe filter with pore size of 0.45 µm and 25 mm diameter to remove any undissolved and suspended particles. Following filtration, RSF was casted into films by pouring concentrated solution in polystyrene petri dish and leaving it for drying at room temperature.

### 2.3. Preparation of PU/RSF Composite Fibres

To produce PU/RSF blended films and fibres, dissolution of these materials in a common solvent is important. In this study, non-aqueous solvent HFIP was used to dissolve PU/RSF composites to prepare dope solution for wet spinning. It is evident from the literature that fluorinated alcohols such as HFIP have a marked potential to dissolve peptides and proteins. Dissolving silk in HFIP produced a stable helical conformation in silk fibroin secondary structure and distinct structural transition from α-helix to β-sheet structure after spinning [37]. Finally, non-aqueous solvent such as methanol denatures native structures of protein and induces formation of β-sheet [38].

Spinning dopes of PU/RSF composites were prepared using HFIP with total polymer concentration of 10% (*w*/*v*). RSF films were weighed according to required weight ratio and dissolved in HFIP using a centrifuge tube at room temperature for 14 h. This was followed by the addition of PU pellets and mixing was continued for 12 h to ensure proper blending. The 100% PU was kept as control and the relative polymer blended compositions were prepared with different PU/RSF weight ratios: 90/10, 75/25, 50/50, 25/75, 10/90 and 0/100. After blending, films were casted by drying overnight in a petri dish. PU/RSF composite fibres were extruded by spinning the dopes in methanol coagulation bath using a syringe pump at a rate of 20 µL/min at room temperature. Next, the fibres are wound using a spool and immersed in the same solution overnight. After rinsing in deionized water, the fibres were dried at room temperature overnight.

### 2.4. FTIR-ATR Spectroscopy

The experiment was performed on Perkin Elmer^®^ Spectrum 100 (PerkinElmer, Waltham, MA, USA), Fourier Transform Infrared (FTIR) spectrometer with Attenuated Total Reflectance (ATR) accessory with Zinc Selenide (ZnSe) crystal plate. The spectra of blends in the range 2200–1100 cm^−1^ with a nominal resolution of 4 cm^−1^ were observed

### 2.5. Thermal Analysis

To determine the thermal stability of the composite, thermogravimetric analysis test was conducted using Mettler Toledo^®^ TGA/DSC (Mettler Toledo, Columbus, OH, USA). The temperature range was set from 30 to 700 °C at a heating rate of 10 °C/min in nitrogen atmosphere.

### 2.6. Tensile Properties

The tensile strength and elongation at break of PU/RSF fibres were measured using Instron universal tensile testing machine (Model 5566, Instron Engineering Corp., Norwood, MA, USA). The fibres were tested at a cross head speed of 50 mm/min in room temperature conditions. A gauge length of 30 mm was set between the grips. Five sample measurements for each blend composition and their average values were calculated.

### 2.7. Scanning Electron Microscopy Analysis

The morphology of the fibres was observed using scanning electron microscopy (SEM, JEOL Model JSM-6490, Tokyo, Japan). The diameter of fibres were obtained using the SEM images. Ten measurements were made at random locations in each sample to calculate the average diameter of fibres.

### 2.8. Transmission Electron Microscopy (TEM)

TEM is significant technique used to study the mesoscale structure of fibres from a smaller volume of sample compared to X-ray diffraction. Selected Area Electron Diffraction (SAED) patterns are scattering from crystals or ordered region to be resolved which can be obtained using TEM. The PU/RSF composite fibres specimens were prepared for TEM by the following method: the fibres were washed and dehydrated in acetone and embedded in epoxy resin (Sigma Aldrich, Saint Louis, MO, USA) and cured at 45 °C for 24 h. One hundred-nanometre-thick transverse sections were cut using Leica Utracut-R ultramicrotome equipped with diamond knife. Finally, cut sections were floated onto 3 mm, 200 mesh copper grids. The dark field images of diffraction patterns from different sections were obtained using JEM-2100F (JOEL, Peabody, MA, USA) equipped with liquid nitrogen cooling system operating at 200 kV.

## 3. Results and Discussions

**Figure 3:**
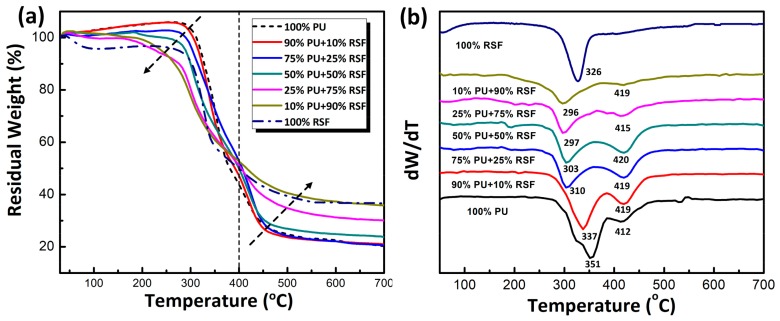
(**a**) Thermogravimetric Analysis (TGA) of PU/RSF blended fibres with different blend ratios; and (**b**) differential thermogravimetric (DTG) curves of PU/RSF blended fibres with different blend ratios.

As shown in Figure 3a, the thermal behaviours of composite fibres are evaluated by thermogravimetric analysis (TGA) at a PU/RSF ratio of 100/0, 90/10, 75/25, 50/50, 25/75, 10/90 and 0/100. Before 400 °C, faster decomposition rate was observed in compositions with higher RSF contents, whereas it was opposite after 400 °C. The faster weight loss of RSF dominant fibres may be attributed to low molecular weight RSF molecules and moisture content. In regard to the increased RSF content, the final residual weights of composites were also increased with more char formation [39]. To further demonstrate the thermal properties of PU/RSF composite fibres, differential thermogravimetric (DTG) curves (Figure 3b) have been made to reveal the possible interactions between two phases. The maximum thermal decomposition temperatures (*T*_max_) of 100% PU matrix were found at 351 °C (peak I) and 412 °C (peak II), which were assigned to hard and soft segments, respectively. Pure RSF showed a *T*_max_ at 326 °C, attributed to the β-sheet crystalline. It is worth noting that the main decomposition peak (peak I) of PU/RSF composite fibres appeared in a range of 296–337 °C, which was lower than pure PU and RSF. It indicated that chemical interactions between these two phases occurred. Obviously, any components from PU and RSF or their degradation products reacted with each other to facilitate the degradation of PU/RSF composites [25]. Thereby, these results suggest the chemical interactions within PU/RSF composites under thermal degradation conditions.

**Figure 4:**
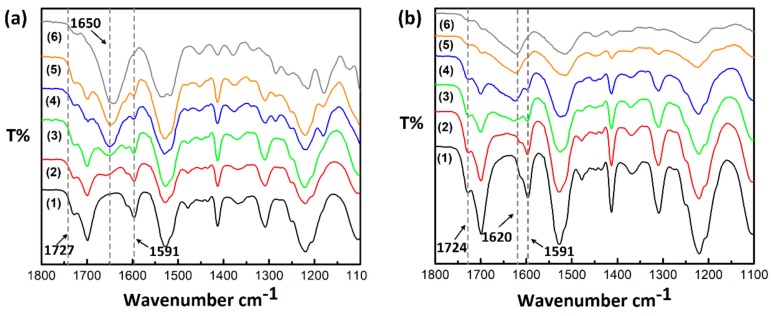
Fourier Transform Infrared (FT-IR) spectra: (**a**) before spinning (film); and (**b**) after spinning (fibres) ((1) PU 100%; (2) PU/RSF 90/10; (3) PU/RSF 75/25; (4) PU/RSF 50/50; (5) PU/RSF 25/75; and (6) PU/RSF 10/90).

The secondary structure of silk fibroin consists of the major conformations including α-helix (silk I) and β-sheet (silk II). FTIR spectra of PU/RSF composite film and fibre are shown in Figure 4. The composites contain 10%, 25%, 50%, 75% and 90% of RSF. The FTIR spectrum of PU ranging from 1720 to 1730 cm^−1^ in the blended films and fibres were attributed to (C=O) carbonyl stretching. The typical absorption band at 1650 cm^−1^ in the amide I region of PU/RSF film is attributed to random coil or α-helix conformation. With regard to the increased concentration of RSF from 10% to 90%, the corresponding peak intensity was also increased. Distinct to PU/RSF films, the composite fibres prepared from methanol coagulation bath were observed with a pronounced peak centred at 1620 cm^−1^, indicating the presence of β-sheets. Shifting of peak from 1650 to 1620 cm^−1^ can be attributed to the conversion of α-helix to β-sheets. Henceforth, the reconstruction of β-sheets in the PU matrix was achieved by wet spinning of fibres using water immiscible solvents. As previously reported [40,41,42], the secondary structure of silk-based materials could be effectively changed by alcoholic solutions. The absorption band at 1720–1730 cm^−1^ (C=O stretching vibration) and 1591 cm^−1^ (benzene ring stretching) are characteristic peaks of polyurethane. Any specific interactions such as strong hydrogen bonding between PU and silk fibroin will result in peak shift or new peak [25,26]. However, the spectra obtained for the 100% PU and PU/RSF blends do not show any peak shift or presence of new peak in this region and suggests that no specific interactions between PU and RSF content. This confirms the PU and RSF components are phase separated and immiscible. It is worth noting that the blending of PU/RSF has not produced any strong interactions between the components under ambient conditions, which was different to DTG results under thermal degradation conditions. In addition, it is evident that the immiscible nature of compositions does not affect the β-sheet confirmation of RSF content in the fibre after spinning.

**Figure 5:**
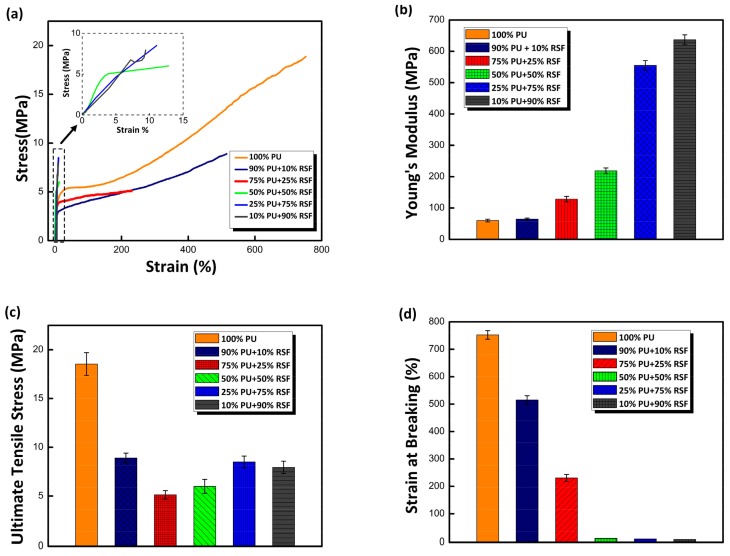
Tensile properties of PU/RSF blended fibres: (**a**) Stress–strain curves of PU/RSF composite fibres. The blend composition of 75/25 exhibits FTSS properties. The stress–strain curves with significantly less strain values are displayed in insets. (**b**) Young’s modulus of PU/RSF composite fibres. The increase in RSF component improved young modulus of fibres significantly (**c**) Ultimate tensile stress of PU/RSF composite fibres. The 100% PU represents highest tensile stress values than PU/RSF blended fibres. (**d**) Strain at breaking (%) of PU/RSF fibres. The increase of crystalline domains in amorphous PU matrix has reduced the strain at break of fibres significantly.

As highlighted in Figure 5, the evaluation of stress–strain curves of composite fibres unveiled the increase in RSF concentration reduced strength and elongation of fibre. Tensile tests of 100% PU fibre produced a mean ultimate tensile stress of 18.8 ± 1.2 MPa compared to 7.9 ± 0.6 MPa of PU/RSF 10/90. The tensile strength of the fibre decreased initially with the increase in RSF component. However, surprisingly when RSF concentration is greater than 25% a slight increase in tensile strength was observed in RSF dominated fibre. Perhaps, this is due to comparatively better orientation of crystalline hard segments at higher concentrations of RSF component [43]. Similarly, strain at breaking of 100% PU was found to be 752% ± 15.7%, in contrast PU/RSF 10/90 produced only 10%. As expected, stiffness of the fibre is increased with the increase in RSF concentration. Remarkably, PU/RSF 10/90 fibre produced mean stiffness (637 ± 15.6 MPa) approximately 10 times higher than 100% PU (60 ± 4 MPa). It is very likely due to the increase in crystalline hard segment of the fibre resulted higher stiffness. Reported data clearly demonstrate that increase in RSF content decreased the tensile strength and improved the stiffness. However, strain at break decreased significantly with increasing RSF content.

The most striking result to emerge from the test is that PU/RSF (75/25) fibre reveals a completely different stress train behaviour from 100% PU fibre. The stress–strain curves start with an elastic region for 100% PU fibre, this region is followed by a plastic region and failure occurs with strain hardening. Twenty-five per cent RSF content in PU matrix resulted in optimum stiffness required to produce a flat tensile curve. As discussed earlier, resultant fibre with 75/25 (PU/RSF) indicates a prominent initial modulus, followed by low post-yield similar to tubuliform silk. Significant extensibility exhibited by the fibre after initial modulus is contributed by PU component. Thereby, increasing RSF concentration gradually reduced elongation of the fibre due to high β-sheet content and produced stiffer fibres. In addition, the loss in mechanical property can also be related with immiscibility and lack of strong interaction between PU and RSF components as reported in FTIR.

The synergistic properties of PU and RSF components is noteworthy because, the composite fibres demonstrated flexibility and displayed varying mechanical properties. Addition of RSF component to PU produced fibres with tuneable mechanical properties with controlled variation of strength, elongation and stiffness. Biopolymers such as PGA, PLA and their copolymers are the popular materials used in biomedicine. However, higher stiffness of these materials limits their utilization in soft tissue engineering [44]. Development of softer and flexible polymer will solve this problem. PU/RSF composite fibres with optimum mechanical properties comparable with soft tissues and flexibility makes them a potential candidate for tissue engineering application.

**Figure 6:**
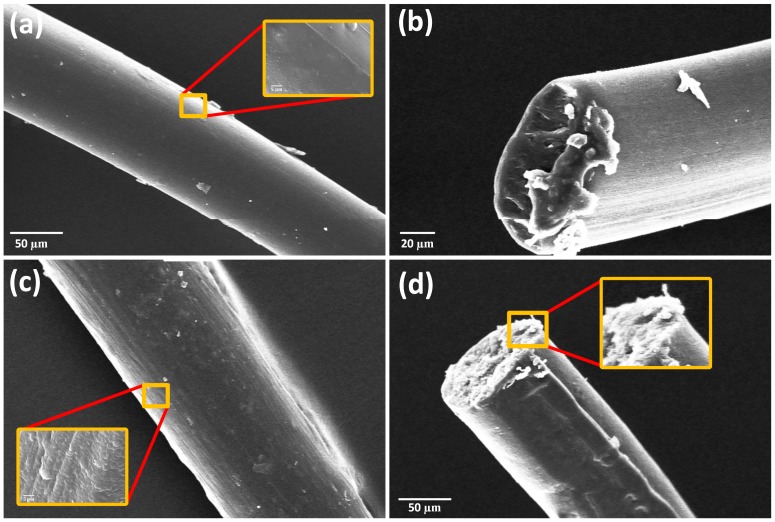
SEM images of PU and PU/RSF composite fibres: (**a**) longitudinal view of PU fibres; (**b**) fractured section of PU fibres; (**c**) longitudinal view PU/RSF (75/25) composite fibres; and (**d**) fractured section view PU/RSF (75/25) composite fibres.

The morphology of PU and PU/RSF composite fibres was analysed using SEM, as shown in Figure 6. We opted to characterize and compare 75/25 PU/RSF on the based on their FTSS properties. The 100% PU fibres displayed a smooth and dense surface with slightly oval cross section. However, after addition of RSF, PU/RSF composite obtained grainy surface structure, which was probably attributed to the microphase separation. It is worth noting that, in fractured area of PU/RSF (75/25), fibrils can be seen protruding from the surface, making a significant contribution to mechanical properties of the fibres [45,46]. At higher concentrations of RSF, the discontinuous RSF fibrils contribute to the increased stiffness of the fibre.

**Figure 7:**
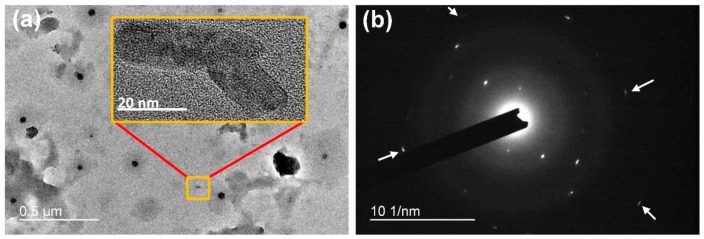
(**a**) Local view of β-sheet crystals in PU/RSF (75/25) composite fibres crystal size ranging from 50 to 450 nm. (**b**) Diffraction pattern obtained from randomly chosen β-sheet crystal in transverse section of fibres PU/RSF (75/25) blended fibres. The patterns show the anisotropic arrangement of the polycrystalline β-sheets. d spacing value of RSF crystals: silk I (Å), 4.4, 4.1, 3.6, and 2.8 Å; silk II (Å), 4.8 and 4.3 Å.

PU/RSF (75/25) fibres were characterized using TEM. The high-resolution images and SAED pattern in Figure 7a,b confirms the presence of RSF crystals in the PU matrix. The SAED patterns obtained from 100% PU do not reveal any crystal diffraction due to its amorphous nature. Interestingly, β-sheet crystals in PU/RSF composite fibres appeared to have a size range from 50 to 450 nm. Figure 7a shows anisotropic distribution of β-sheet crystals with less orientation in the fibre matrix. Inexplicably, diffraction patterns (Figure 7b) show well defined arcs and the projection of the intersheet and interchain directions of β-sheet crystals exhibit anisotropy features similar to the *A. diadematus* egg case silk fibres, as demonstrated by Barghout et al. [10]. However, they also demonstrated transverse sections of *A. diadematus* dragline silk displayed uniform ring characteristics of a uniaxial fibres.

The PU/RSF fibres showed interplanar spacing (d) of 4.8, 4.4, 4.3, 4.1, 3.6, and 2.8 Å. The d spacing values of 4.4, 4.1, 3.6, and 2.8 Å correspond to silk I (type II β turns) structure and the values 4.8 and 4.3 Å are attributed to silk II (antiparallel β pleated sheet) crystalline d spacing. The d spacing attributed to silk II structure 4.8 and 4.3 Å is due to the secondary structure transformation caused by methanol treatment during wet spinning [47]. Our results are reminiscent of previously reported XRD study on PU/SF composites [25], where d spacing values 10, 4.6 and 3.8 Å were attributed to β-sheet crystalline regions. It has been suggested that the overlapping diffraction peaks of PU and RSF components indicated the PU content has no interference in the crystalline region of RSF.

**Figure 8:**
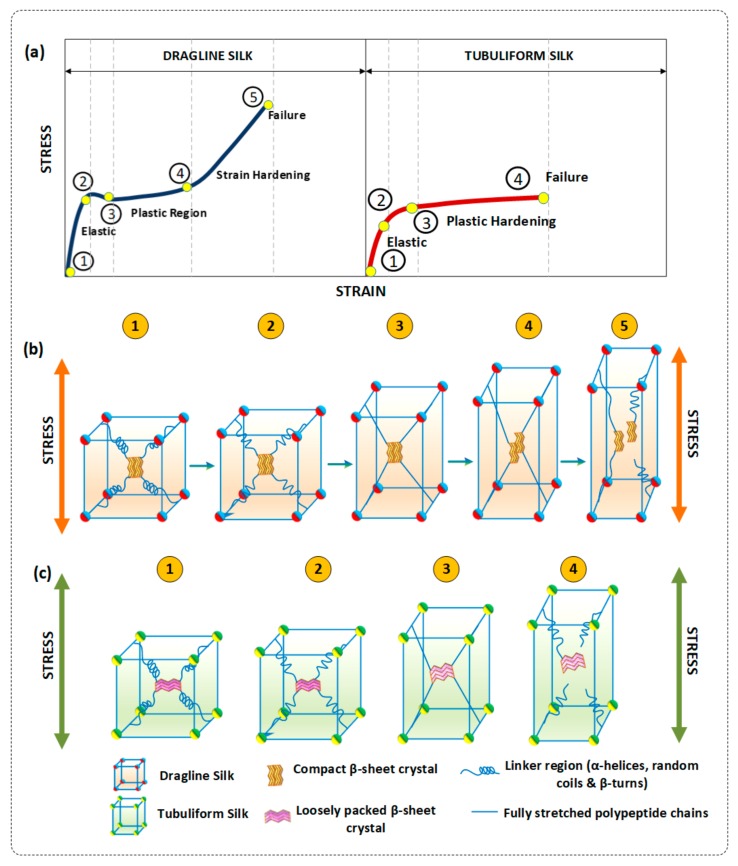
(**a**) Schematic for different stages of deformation in dragline silk and tubuliform silk (Reproduced with permission from [7], 2006 Sage Publications); (**b**) deformation model of dragline silks; and (**c**) deformation model of tubuliform silks.

Based on the earlier experimental studies [9,48] of dragline and tubuliform silk, a mechanical deformation model is presented to explain the FTSS curves. A typical model of stress–strain curves of dragline and tubuliform silks belong to *A. diadematus* spider [7,9]. As shown in Figure 8, deformation model reveals the different stages of deformation of dragline and tubuliform silk under lateral loading. Figure 8b shows the deformation pattern of dragline silk under tension. The slope from 1 to 2 is the initial elastic modulus of the silks. The gradient of the curve falls sharply after yield point (2). In dragline silk, the post yield modulus increases with strain until the final break point at 5. At Stage 1, silk fibres at relaxed state with no tension applied. At Stage 2, fibre experiences high tangent modulus due to stretching causes the hydrogen bond in α-helix and β-turns of the semi-amorphous regions to break. Stage 3 is plateau regime, where the fibres exhibits plastic deformation in this region. This stage is completely attributed to the nanoscale level behaviour of semi-amorphous regions in the silk, where α-helix, β-turns and hidden length of polypeptide chains are uncoiled. Now, the fibre experiences lower stress values despite increase in strain values. At Stage 4, the stress–strain curve enters high stiffness region where the influence of stress shifts from semi amorphous regions to nanoscale β-sheet crystals. The higher stiffness of the β-sheet crystals allows them to sustain larger amount of strain. The β-sheet strands arranged along the uniaxial fibres direction starts to slide with the increase in strain. This stage will last longer if the crystal size is smaller but, in case of larger β-sheet crystals the fibrils will start to break. Finally, at Stage 5, the β strands are completely pulled out and failure occurs at maximum tensile strength of the silk fibres.

Figure 8c shows the deformation of tubuliform silk under lateral loading. In case of tubuliform silk, β-sheet crystals exhibit multiaxial anisotropic properties. It is clear, the role of uniaxial properties exhibited by β-sheet crystals dragline silk to support the load bearing capability of the fibres. Tubuliform silk, which is abundant in short repetitive alanine, serine with voluminous side group (CH_2_OH) and other unevenly repeating amino acid with side chains, produces loosely packed β-sheet which can be easily deformed under stress. Deformation patterns of Stages 1 and 2 in egg case silk is similar to the dragline silk. In Stage 3, the fibres enters the plastic hardening stage, which is a low stiffness region and the deformation switches from semi-amorphous to crystalline region. In contrast to dragline silk, increase in strain causes the transversely arranged and loosely packed β-sheet crystal to disorient from its natural alignment with less resistance to the strain Finally, fibre failure occurs when the fibrils fails to pull β-strands from the anisotropic crystals due to their lower energy. This phenomenon produces failure at plastic hardening region and results in FTSS curve. As a result, after initial modulus, fibres experiences constant stress values despite of increase in strain values.

Crystalline domains in silk are responsible for storage of mechanical energy whereas the amorphous domains dissipate energy. These two main parameters govern the stiffness and toughness of silk respectively [49,50]. Our model for the deformation behaviour of dragline and tubuliform silks shows the influence of ordered (crystalline) and disordered (amorphous) regions in mechanical behaviour of spider silk fibres. Voluminous β-sheets crystal made up of imperfectly repeating amino acid dissipates energy and deforms with less resistance. This lower resistance offered by the β-sheet may contribute to the FTSS behaviour of silk to experience constant stress values.

Additionally, the nanometre size of the structural domains also play an important role in efficient sharing of energy between order between ordered and disordered phases. For higher strength, the β-crystallites must be small enough to ensure a high-volume fraction and on other hand they must be large enough to guarantee a high modulus. The optimum thickness of high modulus layer around the crystal was found to be 5 nm. The increased size of crystals lowers the volume fraction and reduces the elastic modulus of the layer as well as the elongation of the fibres [51].

The spinning of silk by spider or silkworm and artificial spinning methods for silk or other synthetic fibres have significantly different flow dynamics. In natural spinning process, there is a controlled alignment of molecular chains under controlled drawing conditions simultaneously. In wet spinning of regenerated silk, the dope solution was extruded through a die into a chemical coagulant bath where the main alignment of molecules depends on post bath drawing. The coagulant bath causes dehydration and changes the secondary structure along with various spinning conditions affecting the morphology of final fibres. The wet spinning of PU/RSF composite fibres does not offer great control over the β-sheet alignment along the fibres axis and results in the anisotropic random distribution. In PU/RSF composite fibres, β-sheet crystallites are separated by the amorphous region made up of PU chains. Similar to other silk fibres, the β-sheet crystalline domains formed by RSF act as crosslinks between the amorphous region in the PU matrix with hydrogen bonds.

The size of the newly formed crystal like β-sheet domains varies from 50 to 450 nm which is much higher than the optimum level required for better mechanical properties. Hard segment domain size of 100% PU ranges between 3 nm and 10 nm [52]. The 100% PU show better mechanical properties due to their well-defined hard and soft segments. Despite the better mechanical properties, the desirable FTSS behaviour of the composite fibres was achieved with the presence of β-sheet domains formed by RSF component. The SAED patterns from TEM confirms tubuliform silk like anisotropy of crystal regions in the PU/RSF fibres matrix. Even though the chemical composition and mechanical properties of tubuliform silk and silk worm silk are distinctly different, PU/RSF composite fibres imitate microstructure of tubuliform silk by forming anisotropic β-sheet crystals in synthetic polymer matrix. The FTSS behaviour of PU/RSF composite fibres can be associated with the proposed model based on the coinciding factors between tubuliform silk and the composite fibres.

## 4. Conclusions

In summary, a method to produce tubuliform silk like FTSS behaviours is presented by blending PU with RSF at a ratio of 75/25. The secondary structure and crystalline arrangement within RSF are vital in determining the mechanical properties of the fibres. This paper supports the idea of simulating tubuliform silk like anisotropic microstructure in synthetic amorphous polymer matrix to produce FTSS properties. PU/RSF composite fibres were spun using methanol as a coagulant resulted in secondary structure transformation of RSF from α-helix to β-sheets. The conformational changes identified using FTIR confirmed the reconstruction of β-sheet crystals by RSF component in the polymer matrix. Additionally, TEM diffraction patterns of PU/RSF fibres showed good agreement with the multiaxial anisotropic microstructure of *A. diadematus* spider egg case silk. Comparison of dragline and tubuliform silk deformation models demonstrates the relationship between the microstructural arrangement and mechanical behaviour of PU/RSF composites. Such studies are important for understanding the influence of macromolecular assembly in determining fibre properties. Evidently, the composite fibres also display tuneable mechanical properties, which was strongly correlated with RSF contents. It is worth noting that the special property of the tubuliform silk will be very useful for textile applications that require constant pressure at different elongations such as body fitting garments, compression stockings and so on. In addition, tuneable mechanical properties of the composite fibres can be used to produce tailor-made materials for biomedical applications. We are currently now extending this research in investigating the shape memory properties of the composite fibres.
